# Supplementary material for: Can the delayed effects of climatic oscillations have a greater influence on global fisheries compared to their immediate effects?
Source: PLoS One. 2024 Aug 29;19(8):e0307644. doi: 10.1371/journal.pone.0307644 (PMC11361439; doi:10.1371/journal.pone.0307644)
Supplement: S1 Table — (DOCX) [file pone.0307644.s001.docx]

**Supporting Information 1.** Sources of various climate oscillation data.

| **Climatic Oscillation** | **SOURCE** |
| --- | --- |
| NAO | <https://www.cpc.ncep.noaa.gov/products/precip/CWlink/pna/norm.nao.monthly.b5001.current.ascii.table> |
| AMO | <https://psl.noaa.gov/data/correlation/amon.us.long.data> |
| PDO | <https://psl.noaa.gov/data/correlation/pdo.data> |
| IOD | <https://psl.noaa.gov/gcos_wgsp/Timeseries/DMI/> |
| SIOD | <https://www.jamstec.go.jp/virtualearth/general/en/index.html> |
