# Supplementary material for: Can the delayed effects of climatic oscillations have a greater influence on global fisheries compared to their immediate effects?
Source: PLoS One. 2024 Aug 29;19(8):e0307644. doi: 10.1371/journal.pone.0307644 (PMC11361439; doi:10.1371/journal.pone.0307644)
Supplement: S2 Table — The selected items are the ones in bold. (DOCX) [file pone.0307644.s002.docx]

**Supporting Information 2.** Evaluating the influence of climatic fluctuations on the catch rate of three marlin species with Generalized Additive Models (GAMs). The selected items are the ones in bold.

| Striped marlin | | | | | | | | | | | | | | | |
| --- | --- | --- | --- | --- | --- | --- | --- | --- | --- | --- | --- | --- | --- | --- | --- |
| Lag | **NAO** | | | **AMO** | | | **PDO** | | | **IOD** | | | **SIOD** | | |
|  | **Deviance**  **explained (%)** | **GCV** | **AIC** | **Deviance**  **explained (%)** | **GCV** | **AIC** | **Deviance**  **explained (%)** | **GCV** | **AIC** | **Deviance**  **explained (%)** | **GCV** | **AIC** | **Deviance**  **explained (%)** | **GCV** | **AIC** |
| 0 | - | - | - | **86.7*** | **5.25*** | **50.21*** | - | - | - | - | - | - | **99.7*** | **0.88*** | **12.98*** |
| 1 | - | - | - | 9.54 | 10.74 | 64.16 | - | - |  | **42.5*** | **6.83*** | **58.74*** | 26.4 | 8.73 | 61.69 |
| 2 | - | - | - | - | - | - | **91.4*** | **4.61*** | **46.49*** | 20.4 | 9.44 | 62.62 | - | - | - |
| 3 | **76.3*** | **8.26*** | **56.43*** | 75.2* | 9.01* | 57.21* | 40.8 | 7.52 | 59.74 | - | - |  | 30 | 9.93 | 62.79 |
| 4 | - | - | - | - | - | - | 11.4 | 10.52 | 63.92 | 38 | 8.51 | 60.48 | 15 | 10.1 | 63.42 |
| 5 | - | - | - | 65.3 | 9.04 | 59.17 | - | - | - | 26.7 | 9.95 | 62.94 | 15.9 | 9.98 | 63.29 |
| 6 | 44.5 | 9.19 | 61.37 | 28.7 | 12.46 | 61.36 | 33.3 | 8.41 | 61.11 | - | - | - | - | - | - |
| 7 | - | - | - | - | - | - | - | - | - | - | - | - | - | - | - |
| 8 | - | - | - | - | - | - | 41.9 | 8.46 | 60.79 | 31.9 | 8.68 | 61.19 | - | - | - |
| Blue marlin | | | | | | | | | | | | | | | |
| Lag | **NAO** | | | **AMO** | | | **PDO** | | | **IOD** | | | **SIOD** | | |
|  | **Deviance**  **explained (%)** | **GCV** | **AIC** | **Deviance**  **explained (%)** | **GCV** | **AIC** | **Deviance**  **explained (%)** | **GCV** | **AIC** | **Deviance**  **explained (%)** | **GCV** | **AIC** | **Deviance**  **explained (%)** | **GCV** | **AIC** |
| 0 | 9.4 | 46.71 | 81.81 | 33.3* | 34.39* | 78.13* | 55.6**~** | 28.99**~** | 75.47**~** | - | - | - | **94.9**** | **10.11**** | **57.06**** |
| 1 | 19.8 | 45.21 | 81.21 | 23.4 | 39.51 | 79.79 | - | - | - | - | - | - | 70.1*** | 15.4*** | 68.49*** |
| 2 | - | - | - | 32 | 40.92 | 79.85 | 41.7 | 38.87 | 78.92 | - | - | - | 78.7 | 35.31 | 73.27 |
| 3 | - | - | - | - | - | - | **74.2*** | **21.27*** | **70.91*** | - | - | - | 49.1 | 33.37 | 77.14 |
| 4 | **43.8~** | **43.29~** | **79.71~** | - | - | - | 62.3** | 19.41** | 71.27** | 45* | 28.37* | 75.82* | - | - | - |
| 5 | 22 | 40.23 | 80.01 | **96.6~** | **23.05~** | **57.01~** | 35 | 33.51 | 77.82 | 32.4**~** | 34.86**~** | 78.29**~** | 22.2 | 44.66 | 81.02 |
| 6 | 24.9 | 38.73 | 79.55 | 12.7 | 44.99 | 81.35 | - | - | - | 42.3* | 34.66* | 77.86* | - | - | - |
| 7 | 9.12 | 46.85 | 81.84 | 64.7* | 31.61* | 75.32* | - | - | - | **66.3*** | **23.11*** | **72.61*** | 38.9* | 31.51* | 77.08* |
| 8 | - | - | - | 58**~** | 54.94**~** | 80.01**~** | - | - | - | - | - | - | 11.1 | 45.82 | 81.57 |
| Silver marlin | | | | | | | | | | | | | | | |
| Lag | **NAO** | | | **AMO** | | | **PDO** | | | **IOD** | | | **SIOD** | | |
|  | **Deviance**  **explained (%)** | **GCV** | **AIC** | **Deviance**  **explained (%)** | **GCV** | **AIC** | **Deviance**  **explained (%)** | **GCV** | **AIC** | **Deviance**  **explained (%)** | **GCV** | **AIC** | **Deviance**  **explained (%)** | **GCV** | **AIC** |
| 0 | - | - | - | 18.8 | 4.11 | 52.65 | **96.8*** | **1.41*** | **7.11*** | 21.4 | 3.98 | 52.26 | **99.6*** | **0.32*** | **2.98*** |
| 1 | - | - | - | 14.7 | 4.32 | 53.24 | - | - | - | 79.1* | 1.82* | 41.11* | 80.2* | 1.91* | 41.24* |
| 2 | - | - | - | 29.6 | 4.14 | 52.37 | - | - | - | - | - | - | 16.1 | 4.24 | 53.03 |
| 3 | 9.33 | 4.59 | 53.97 | - | - | - | 26.9~ | 3.71~ | 51.39~ | - | - | - | 32.5~ | 3.42~ | 50.44~ |
| 4 | 18 | 4.15 | 52.77 | - | - | - | 93.1*** | 0.49*** | 26.28*** | 9.34 | 4.59 | 53.97 | - | - | - |
| 5 | - | - | - | - | - | - | 38.6* | 3.11* | 49.29* | **96.8*** | **2.14*** | **28.36*** | 59.8* | 2.56* | 46.39* |
| 6 | - | - | - | - | - | - | 9.99 | 4.56 | 53.88 | 48.8 | 3.61 | 50.16 | - | - | - |
| 7 | 19.7 | 4.07 | 52.52 | 61.9~ | 3.18~ | 47.99~ | - | - | - | - | - | - | 15.1 | 4.31 | 53.18 |
| 8 | **73.5~** | **2.78~** | **45.32~** | **88.7*** | **1.95*** | **38.17*** | 24.2 | 3.84 | 51.82 | - | - | - | - | - | - |

Significance levels: *** 0.001, ** 0.01, * 0.05, ~ 0.1.
