# Supplementary material for: Can the delayed effects of climatic oscillations have a greater influence on global fisheries compared to their immediate effects?
Source: PLoS One. 2024 Aug 29;19(8):e0307644. doi: 10.1371/journal.pone.0307644 (PMC11361439; doi:10.1371/journal.pone.0307644)
Supplement: S1 Fig — (DOCX) [file pone.0307644.s003.docx]

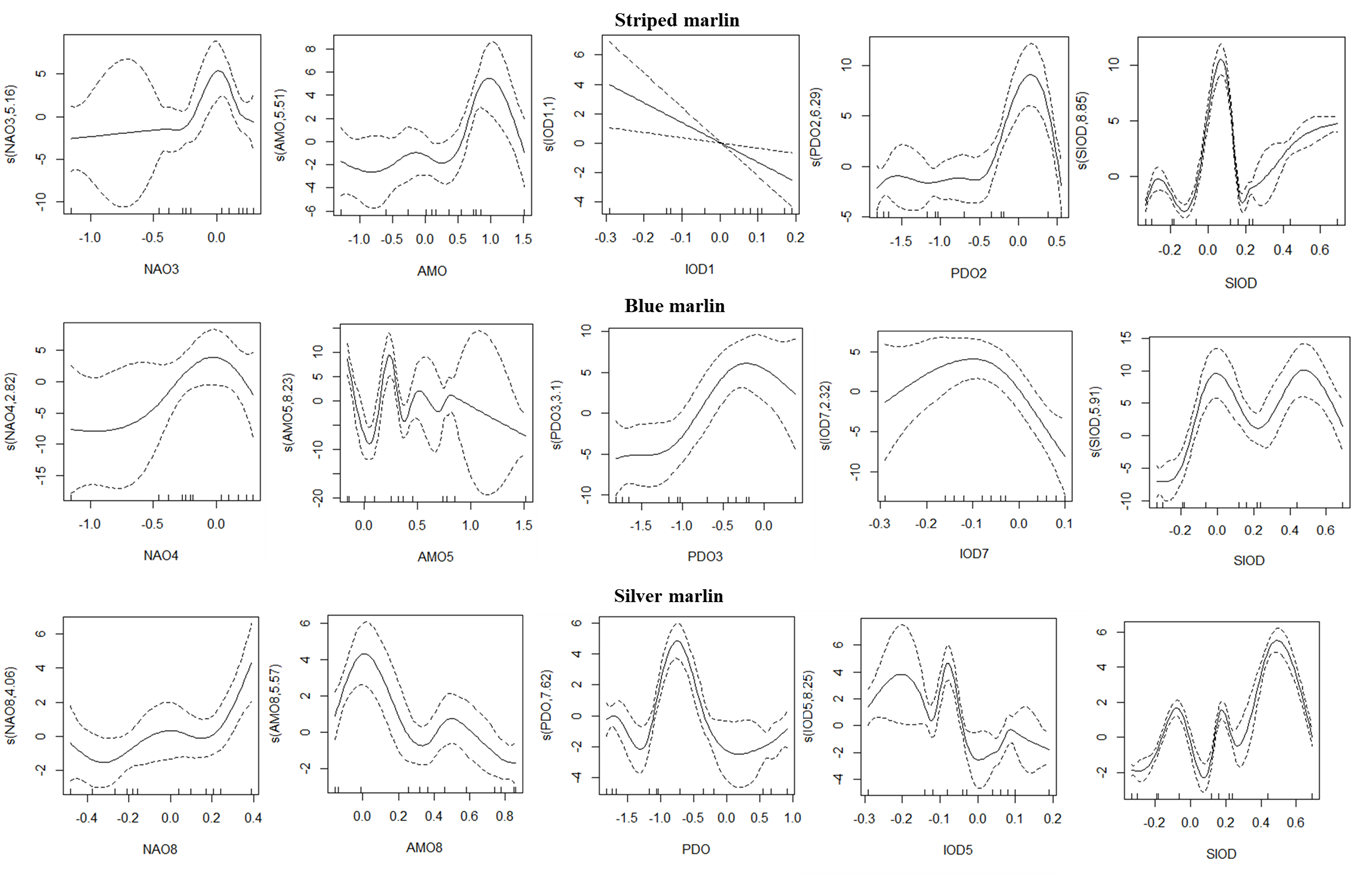


**Supporting Information 3.** Partial effect plots of the marlins with the most significant lag of each climatic oscillation.
