# Supplementary material for: Can the delayed effects of climatic oscillations have a greater influence on global fisheries compared to their immediate effects?
Source: PLoS One. 2024 Aug 29;19(8):e0307644. doi: 10.1371/journal.pone.0307644 (PMC11361439; doi:10.1371/journal.pone.0307644)
Supplement: S3 Fig — (DOCX) [file pone.0307644.s005.docx]

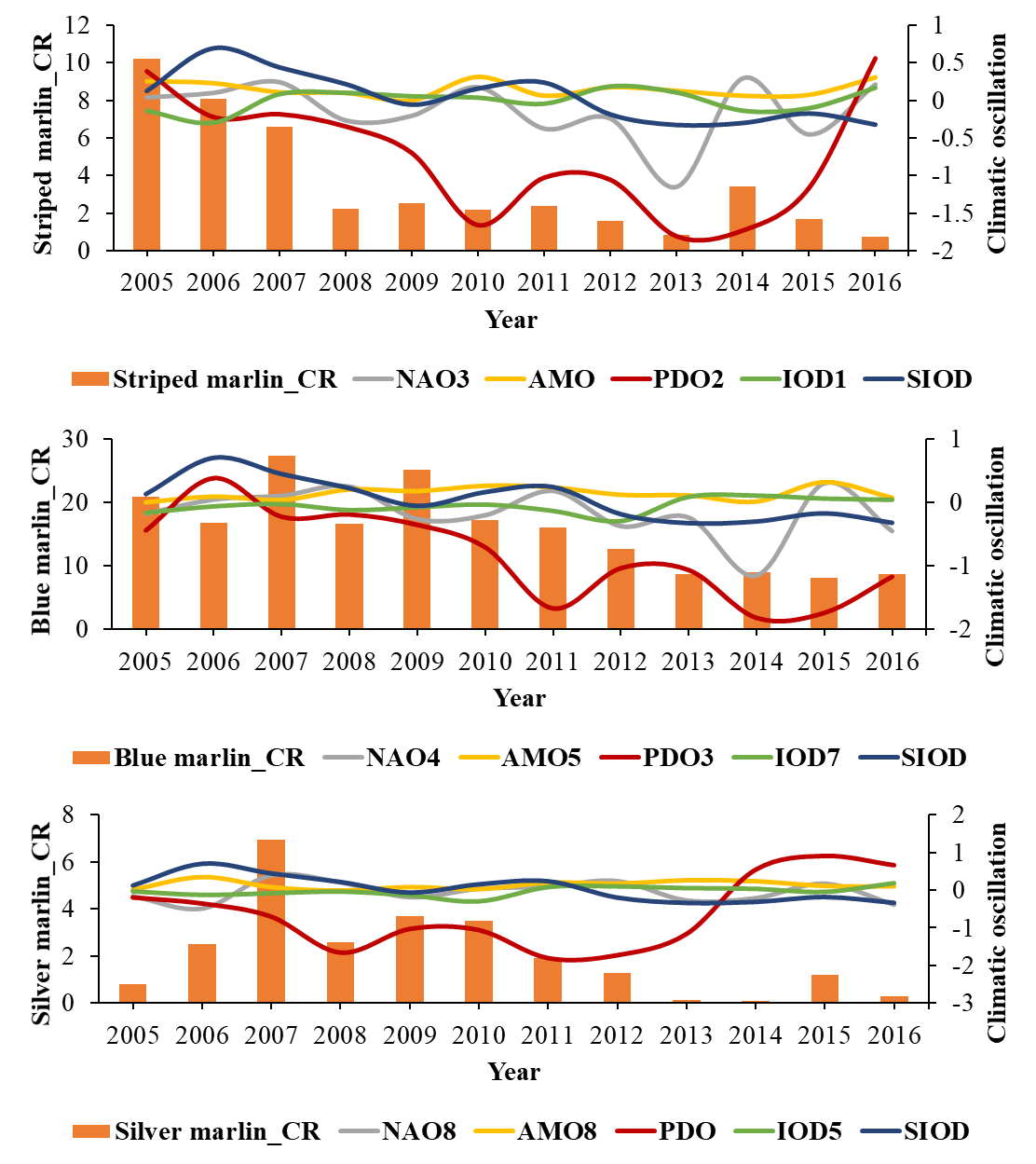


**Supporting Information 5.** Annual fluctuations in the capture of three marlin species over the research period with the chosen lag for each climatic oscillation.
