# Supplementary material for: Can the delayed effects of climatic oscillations have a greater influence on global fisheries compared to their immediate effects?
Source: PLoS One. 2024 Aug 29;19(8):e0307644. doi: 10.1371/journal.pone.0307644 (PMC11361439; doi:10.1371/journal.pone.0307644)
Supplement: Attachment — Submitted filename: Respond to Reviewers.docx [file pone.0307644.s007.docx]

**Comment-wise Replies to Reviewer 1:**

| **Teleconnections between oscillations and fishery yield is not new .. what exactly the novelty the authors tries to bring is to be elaborated** | We thank the reviewer for the comment.  Climatic oscillations through teleconnections present a favorable occasion to examine and assess the impact of microhabitat changes on the distribution of fish. Despite our extensive knowledge, significant gaps still exist, especially regarding the consequences of climate change on the intricate food web that sustains marlin populations and their fisheries. It is uncertain whether climate change will affect the relationships between marlin fishing and the structure and functioning of the Atlantic Ocean ecosystem. By identifying changes in the distribution of marlin, we can enhance our comprehension of the habitat needs of crucial marine resources and improve our future management of marlin fisheries. There is a lack of comprehensive data on climate variability and its effects on marlin resources in the Atlantic Ocean. Undoubtedly, comprehending the impact of the oceanic environment on the distribution of tuna species is a crucial prerequisite for implementing ecosystem-based management of fisheries, which is progressively becoming a fundamental necessity in management policy. Therefore, the novelty of this research lies in the fact that it has tried to investigate the impact of climatic oscillations on marlin availability in the Atlantic Ocean. There has been no previous research that has examined this aspect and our study is the first one to also find the association of three marlin species whose distribution or catch rate was found to be influenced by three oscillations despite being located in the same ocean, that also points to the species-specific responses to climatic changes. |
| --- | --- |
| **L96 onwards must be redrafted ..her it is the summary of work done /future studies etc .. but what expected is the research gap and stating the objectives precisely.. say (i) .. (ii)** | The section has been re-drafted as suggested by the reviewer and the research objectives and gaps have been addressed now.  **Line 131 – 175 (File name: Manuscript)** |
| **L146 and L192 what is the difference ? here wavelet coherence is used in the latter case . how it is different from correlation ? why you need to use coherence ?** | We thank the reviewer for the comment.  Wavelet analysis is a widely employed technique for analyzing time series data. It is a method that enables analysis of periodic components and their temporal variations by representing the data in a time–frequency space. The technique computes a wavelet coefficient for each location in the dataset by measuring the correlation between the observed signal (in this case, the selected climatic index) and the wavelet. The outcome is a matrix containing wavelet coefficients for every possible combination of scale (representing the frequency of the wavelet) and translation (indicating the shift of the wavelet along the signal). Wavelets provide the advantage of simultaneously examining many scales. The wavelet transform breaks down a time series into its constituent time, frequency, and power components, which can be seen in a three-dimensional space using the wavelet power spectrum (WPS) plot. In a WPS plot, the x-axis represents the time series, while the y-axis represents the contribution of frequencies, which is indicated by the term "period". The term "power" refers to the measure of the amount of variation present in a time series at a specific wavelet. The WPS assesses the aspects of the signal that are influential and contributory, as well as those that are less important. Wavelet coherence was used to investigate environmental variables with the greatest predictive power for analysis of fisheries productivity. The wavelet coherence was the mean coherence within the significant coherence zone, so a larger coherence indicated a more significant contribution of the variable to explain variability in response variables. These plots were used to identify periods of greatest correlation (represented by red points in the figures). On the other hand, one of the most commonly used methodologies to identify potential relationships between variables in climate research is correlation, with or without a lag (or time delay). Such correlation (or linear regression) approaches, despite being useful for identifying potential relationships between variables, do not imply causation. A significant correlation simply means that there is a relationship, or synchronous behavior, between two variables without explicitly confirming a causal link between the two. Thus, as a preliminary step, the cross-correlation between different variables and marlin catch rate was computed in the present study. The same methodology has been followed by **Adarsh et al., 2024**. Wavelet coherence combines the advantages of wavelet analysis and Pearson correlation, allowing for searching for correlations that vary over frequency and time.  **Ref.** Adarsh, S., Fathima, S. and Arunkumar, R., 2024. Multiscale teleconnection analysis of rainfall patterns over Calicut, India using wavelet coherence. Journal of Earth System Science, 133(1), pp.1-16.  Ma, S., Huse, G., Ono, K., Nash, R.D., Sandø, A.B., Nedreaas, K., Sætre Hjøllo, S., Sundby, S., Clegg, T., Vølstad, J.H. and Kjesbu, O.S., 2024. Recruitment regime shifts and nonstationarity are widespread phenomena in harvestable stocks experiencing pronounced climate fluctuations. Fish and Fisheries, 25(2), pp.320-348.  Mathews, J. and Czaja, A., 2024. Oceanic maintenance of atmospheric blocking in wintertime in the North Atlantic. Climate Dynamics, pp.1-14. |
| **Basically wavelet coherence is used to find association between catch rate and oscillation .. what is the logic in taking the series of catch rate and its wavelet analysis ?how you physically justify it ? oscillation is a process /physical phenomenon** | We thank the reviewer for the comment.  Wavelet analysis uses a set of functions locally defined in both time and frequency domains. Wavelet analysis performs a time-frequency analysis of the signal, which permits the estimation of the spectral characteristics of the signal as a function of time and then the identification of different periodic components and their time evolution all along the time series. In other words, transient dynamics or gradual changes of the periodic components of the signal can be detected. Furthermore, cross-wavelets and phase analysis generalize these possibilities to the analyses of dependencies between two signals. Wavelet coherence, however, can help resolve relationships when time lags confound correlation. Two variables are coherent if they exhibit correlated oscillations and consistent phase differences through time, even if the variables are phase-lagged or anti-phase (in our study these two variables are the marlin catch rate and oscillation). Consistency of phase differences is important because it is more likely to imply a causal relationship that is not due to chance. Furthermore, identification of the type of phase relationships can help inform the potential drivers. Like other wavelet approaches, wavelet coherence also accommodates complex temporal autocorrelation structures, another common property of fisheries time series that confound correlation tests. Therefore, we used wavelet coherence analyses to determine whether marine marlin fishery catches were coherent in the study region during the time considered, and to determine the distribution of phase relationships between fishery catches.  **Ref.** Sanz-Fernández, V., Gutiérrez Estrada, J.C., Queirolo Palma, D. and Hormazábal Fritz, S., Climatic and Oceanographic Variability Associated with Historical Landings of Blackspot Seabream in the Strait of Gibraltar: Wavelet-Based Analysis. Available at SSRN 4721183.  Ong JJL, Walter JA, Jensen OP, Pinsky ML. Global hotspots of coherent marine fishery catches. Ecol Appl. 2021 Jul;31(5):e02321. |
| **Also some more details on wavelet implementation is required if the above one is properly justified** | We thank the reviewer for the comment.  More details on wavelet implementation have now been added in the revised manuscript like the responses above.  **Line 262 – 312** |
| **Other than being two time series, how you physically justify using the analysis for catch rate ?..the relation is more statistical than physical ?** | We thank the reviewer for the comment.  Understanding the relative effects of the array of factors affecting population dynamics is a primary objective of fisheries science. It is widely accepted that fishing activity modifies population demography and structure, stock sizes and trophic interactions. Climate induced variability is also a strong driver of change in fish populations. Research has focused on associations between climatic process, fishing resources and effects on the egg and larval phases when these early life stages of studied species are highly sensitive compared to later (juvenile and adult) life stages. The influence of climate variability on fish populations can be classified on three different types: direct effects on the survival of pre-recruits or larvae; indirect effects on spawning stock biomass (by changing seasonal reproduction cycle; and mixed effects – a combination of direct and indirect effects. Studies on climate driven effects over fisheries span over a century.  Analysing time series through wavelet or prediction models have been employed to identify the potential cause of environmental impacts on marine fishing resources. The establishment of connections between environmental data and fish stock characteristics or parameters, as well as the integration of these findings into assessments, should be grounded in a comprehensive comprehension of the ecology of the system or region being examined. Furthermore, it should offer insight into the mechanisms elucidated by statistical correlations. The process of determining the causes of variations in landings is intricate, leading to numerous potential ideas to elucidate observed patterns. By employing various analytical methodologies or statistical models, researchers can generate many hypotheses to explain landing patterns. This can lead to a wide range of potential models, many of which may effectively fit the data. Hence, it is imperative to make progress in the assessment of statistical models by employing a model selection technique that emphasizes the evaluation of the models' "predictive" capacities rather than their performance in fitting the data.  Multiple authors concur that marine species have the potential to adapt to climate oscillations by altering their phenology, abundance, distribution, and recruitment. Several studies have examined how climatic cycles affect the abundance and captures of species that are the focus of fisheries. Hence, it is crucial to assess if climate oscillations lead to variations in the abundance and catchability of commercially valuable species, thereby influencing fish market prices by affecting the supply of fishing goods, that can be only done when the catch rate is analysed in junction with the ambient environment of the fish.  **Ref.** Baptista, V. and Leitão, F., 2014. Commercial catch rates of the clam Spisula solida reflect local environmental coastal conditions. Journal of Marine Systems, 130, pp.79-89.  Vânia, B., Ullah, H., Teixeira, C.M., Range, P., Erzini, K. and Leitão, F., 2014. Influence of environmental variables and fishing pressure on bivalve fisheries in an inshore lagoon and adjacent nearshore coastal area. Estuaries and coasts, 37, pp.191-205. |
| **How the joint effect of multiple oscillations influence the results ? to be analyzed and explained** | We thank the reviewer for the comment.  The joint effect of pairs of climatic oscillations has been analysed (Table 3) and explained in detail as per the suggestions.  **Line 314,326, 494-504, 715-767** |
| **Conclusion is more generic .. quantitative information should be added and revised** | We thank the reviewer for the comment.  The conclusion has been revised and restructured as per the suggestions.  **Line 802-824** |

**Comment-wise Replies to Reviewer 2:**

| **Line 17-18:**  **This part is unclear. in the first lines of the abstract it has been stated that the study will examine catch rates in the Atlantic Ocean, while here are reported results from Indian Ocean. Please consider modify either this sentence or the previous ones.** | We would like to thank the reviewer for valuable comments.  Various climate oscillations from different oceans have an impact on the Atlantic Ocean through teleconnection. Teleconnection is a climate phenomenon where weather patterns in one region are influenced by conditions in distant ones, facilitated by large-scale atmospheric or oceanic processes.  For instance, the Indian Ocean dipole (IOD) impacts atmospheric circulation patterns such as the Walker and Hadley circulations, affecting global weather conditions, including Atlantic hurricane development. Changes in sea surface temperatures propagate through ocean currents, indirectly affecting Atlantic region weather patterns and climate phenomena.  The Subtropical Indian Ocean Dipole (SIOD) significantly impacts the Atlantic Ocean through variations in sea surface temperatures, affecting atmospheric circulation patterns, Walker circulation, and precipitation patterns. These anomalies can affect tropical cyclone development, affecting the Atlantic basin and surrounding areas, and playing a role in global climate systems beyond the Indian Ocean.  **Ref:** An S-I, Wang C, Mechoso CR. Teleconnections in the Atmosphere. In: Mechoso CR, ed. Interacting Climates of Ocean Basins: Observations, Mechanisms, Predictability, and Impacts. Cambridge University Press; 2020:54-88.  Prior research has also demonstrated the significance of teleconnections in numerous cases.  **Ref:** Zhang, L. and Han, W., 2021. Indian ocean dipole leads to Atlantic Niño. Nature Communications, 12(1), p.5952.  Báez, J.C., Czerwinski, I.A. and Ramos, M.L., 2020. Climatic oscillations effect on the yellowfin tuna (Thunnus albacares) Spanish captures in the Indian Ocean. Fisheries Oceanography, 29(6), pp.572-583.  The original study focused exclusively on marlin species found in the Atlantic Ocean. Nevertheless, due to the aforementioned reasons, authors also included the use of IOD and SIOD to examine any potential influence of teleconnections on the catch rates of Atlantic Ocean marlin species. |
| --- | --- |
| **Line 45** | Revised as per the suggestion.  **Line 66 (File name: Manuscript)** |
| **Line 97: Please stated here which species and why have been selected** | We would like to thank the reviewer for valuable comments.  Three marlin species were included in the present study and are as follows:  1. Striped marlin - *Kajikia audax*, 1887;  2. Blue marlin - *Makaira nigricans*, 1802;  3. Silver marlin - *Kajikia albida*, 1860  Two species reasons are there behind selecting these species only and are as follows:  1. The specific rationale for choosing only these three marlin species was based on the fishery data only provided by the Overseas Fisheries Development Council of Taiwan, which exclusively consisted of data pertaining to these three marlin species.  2. These three marlin species are vital species in the Indian Ocean, playing a crucial role as apex predators, regulating prey populations, supporting commercial and recreational fishing industries, and generating revenue through tourism. They also symbolize maritime heritage and local traditions.  **Line 131-150 (File name: Manuscript)** |
| **Line 107-109: This sentence seems bit awkward to me. Please consider rephrasing.** | We would like to thank the reviewer for valuable comments.  Authors added some important information also, while rephrasing this line.  “Despite these benefits, no studies have yet comprehensively examined the effects of climatic oscillations and ocean conditions on the catch rates and distribution of marlins in the AO (Mondal et al., 2023). In addition, there are concerns about the stock condition of marlins, in the Atlantic Ocean, as there are indications of overfishing and a decrease in population numbers. As per the International Commission for the Conservation of Atlantic Tunas (ICCAT), the populations of both blue and striped marlins have been excessively exploited and are currently below levels that can be sustained in the long term. Blue marlin populations are significantly reduced due to heavy fishing pressure from commercial longline fisheries, primarily targeting tuna and swordfish, often caught incidentally as bycatch. Hence, in order to guarantee the long-term conservation of fishery resources, additional investigation is necessary to ascertain the impact of climatic oscillations on marlin fisheries due to the ecological and economic significance of marlins.”  **Line 151-163 (File name: Manuscript)** |
| **Line 111-112: This is a repetition of what reported in line 96. I would suggest rephrasing while expanding further the meaning of this study. In my opinion this could benefit the entire MS** | The authors omitted this sentence, as it had previously been stated in line 96 of the previous version of the manuscript. |
| **Line 126-128: I'm not sure if this sentence is necessary. Maybe it could be removed** | We would like to thank the reviewer for valuable comments.  The authors omitted these sentences as per the reviewer suggestion. |
| **Line 132: which resolution was used? one year? Please add it** | Yearly temporal resolution and stated in the revised manuscript.  **Line 190-191 (File name: Manuscript)** |
| **Line 262: The additional information that are reported in table s2 with respect to table 2 should be mentioned.** | We would like to thank the reviewer for valuable comments.  Information is now added as per the reviewer suggestions.  **Line 385-417 (File name: Manuscript)** |
| **Line 384-385: In which way? Please discuss it further** | We would like to thank the reviewer for valuable comments.  Authors stated additional information as follows:  “Overall, our findings regarding the delayed effects of climatic oscillations on catch rates are supported by those of a study on Pacific billfish (Haulsee et al., 2022). This study noted that the effects of climate-scale oscillations, on the presence of billfish may be delayed over time. Another study shown that the consequences of delayed climatic oscillations had a greater effect on the Indian Ocean marlin species compared to immediate impacts (Mondal et al., 2023). Therefore, our current findings about the delayed effects of climatic oscillations, which are more significant than immediate impacts, are consistent with previous research.”  This information is added now to the revised manuscript.  **Line 547-555 (File name: Manuscript)** |
| **Line 453-454: Couldn't it be related to diferent life-history traits of the three marlins? For example overall longevity, TL50 or simply growth rate?** | We would like to thank the reviewer for valuable comments.  This can be related to the life-history traits also. Authors rephrased the sentence and stated additional information as follows:  “Nevertheless, this analysis unveiled divergent patterns in the distributions of the three marlin species, as well as the influence of climate oscillations on various life phases. However, this analysis revealed contrasting trends regarding the distributions of the three marlin species. Since this was the preliminary investigation, the authors solely focused on identifying the delayed effects of climatic oscillations. In the subsequent phase, authors will endeavours to determine the influence of climatic oscillations and their delays on various life phases of marlin species, given that the findings of the preceding investigation have corroborated the current hypothesis.”  This information is added now to the revised manuscript.  **Line 646-652 (File name: Manuscript)** |
|  |  |
| **Table 1:** **I'd suggest to add "(years)"** | Added as per the reviewer suggestion. |
| **Table 2: Please describe every acronym** | Acronym description is added now. |
| **Table 3: Please see comment above** | Acronym description is added now. |
| **Figure 3: Please increase the overall text visibility** | New figure with higher resolution is provided now. |
