# Supplementary material for: Can the delayed effects of climatic oscillations have a greater influence on global fisheries compared to their immediate effects?
Source: PLoS One. 2024 Aug 29;19(8):e0307644. doi: 10.1371/journal.pone.0307644 (PMC11361439; doi:10.1371/journal.pone.0307644)
Supplement: Attachment — Submitted filename: Respond to Reviewers.docx [file pone.0307644.s008.docx]

**Comment-wise Replies to Reviewer 1:**

| **Comments** | **Replies** |
| --- | --- |
| 1. The use of wavelets etc. the justification should be supported by citing some recent references in the main text | We would like the thank the reviewer for this important comment. Following references are added now.  1. Lee, M.A., Mondal, S., Wu, J.H., Huang, Y.H. and Boas, M. Cyclic Variation in Fishing Catch Rates-Influenced by Climatic Variability in the Waters Around Taiwan. Journal of Taiwan Fisheries Society, 49(2), pp.113-125, 2022,  <https://doi.org/10.29822/JFST.202206_49(2).0005>.  **Ref 36**.  2. Yadav, V.K., Jahageerdar, S. and Adinarayana, J. Modeling framework to study the influence of environmental variables for forecasting the quarterly landing of total fish catch and catch of small major pelagic fish of North-West Maharashtra coast of India. National Academy Science Letters, 43(6), pp.515-518, 2020,  <https://doi.org/10.1007/s40009-020-00922-2>.  **Ref 63**.  3. Raubenheimer, C. and Phiri, A. The impact of climate change and economic development on fisheries in South Africa: a wavelet-based spectral analysis. Humanities and Social Sciences Communications, 10(1), pp.1-11, 2023,  <https://doi.org/10.1057/s41599-023-02408-0>.  **Ref 48**.  4. Lan, K.W., Wu, Y.L., Chen, L.C., Naimullah, M. and Lin, T.H., 2021. Effects of climate change in marine ecosystems based on the spatiotemporal age structure of top predators: A case study of bigeye tuna in the Pacific Ocean. Frontiers in Marine Science, 8, p.614594, 2021,  <https://doi.org/10.3389/fmars.2021.614594>.  **Ref 33**. |
| 2. Similarly, the wavelet application in this catches ..is it completely novel ? else to be supported with past studies in the main text | We would like the thank the reviewer for this important comment. Following references are added now.  1. Wu, Y.L., Lan, K.W., Evans, K., Chang, Y.J. and Chan, J.W. Effects of decadal climate variability on spatiotemporal distribution of Indo-Pacific yellowfin tuna population. Scientific Reports, 12(1), p.13715, 2022,  <https://doi.org/10.1038/s41598-022-17882-w>.  **Ref 62**.  2. Lan, K.W., Chang, Y.J. and Wu, Y.L. Influence of oceanographic and climatic variability on the catch rate of yellowfin tuna (*Thunnus albacares*) cohorts in the Indian Ocean. Deep Sea Research Part II: Topical Studies in Oceanography, 175, p.104681, 2020,  <https://doi.org/10.1016/j.dsr2.2019.10468>.  **Ref 34**. |
| 3. Qn 4 need more clarity .. catche rate is just a time series, while AMO etc are a physical process .. how the application of wavelet on a simple time series is justifiable ? for example if the catche series shows a periodicity of 30 .. what does it mean ?  **“Basically wavelet coherence is used to find association between catch rate and oscillation .. what is the logic in taking the series of catch rate and its wavelet analysis ?how you physically justify it ? oscillation is a process /physical phenomenon”** | We would like the thank the reviewer for this important comment.  **(A) Catch rate is just a time series, while AMO etc. are a physical process, how you physically justify it ? oscillation is a process /physical phenomenon**  **Ans.** The catch rate, while considered as a time series, may appear separate from physical phenomena such as the Atlantic Multidecadal Oscillation (AMO). However, there is a compelling rationale for examining them in conjunction, as environmental conditions, which are influenced by physical processes (climatic oscillations), can have a substantial effect on biological and ecological phenomena. **Physical Justification can be as follows:**  ***1. Environmental Influence on Marine Life:*** Any physical process like climatic oscillations involving changes in sea surface temperature, salinity, and ocean currents, impacts marine ecosystems by altering habitat conditions, affecting species distribution, breeding, feeding, and migration patterns. Changes in water temperature and nutrient availability can affect fish populations, affecting catch rates **(Lee et al., 2022, Lan et al., 2020)**.  ***2. Climate-Ocean Interactions:*** Climate oscillations, are influenced by intricate interactions between the atmosphere and ocean, leading to weather changes and subsequently affecting oceanographic conditions. These changes are highly influenced by fish populations, affecting catch rates **(Wu et al., 2022, Yadav et al., 2020)**.  In summary, the analysis of catch rates alongside physical processes like the AMO and other climatic oscillations is justified due to the significant impact of these oscillations on marine ecosystems. The integration of fishery time series data with climate oscillations through statistical and mechanistic approaches provides a robust framework for understanding and predicting these interactions.  **Brief description is added to the discussion section as follows:**  “The catch rate, a time series, may seem separate from physical phenomena like the Atlantic Multidecadal Oscillation (AMO). However, they are interconnected due to their significant impact on marine ecosystems. Changes in sea surface temperature, salinity, and ocean currents alter habitat conditions, affecting species distribution, breeding, feeding, and migration patterns. Changes in water temperature and nutrient availability can affect fish populations, affecting catch rates. Additionally, climate oscillations, influenced by atmospheric and ocean interactions, lead to weather changes and oceanographic conditions, which are highly influenced by fish populations. Integrating fishery time series data with climate oscillations provides a robust framework for understanding and predicting these interactions.” **Line 796-805**  **(B) The application of wavelet analysis to a simple time series is justifiable for several reasons:**  1. Wavelet transforms offer time-frequency localization, allowing analysis of signal frequency changes over time, particularly useful for non-stationary fishery time series data, identifying transient features **(Raubenheimer & Phiri, 2023)**.  2. Wavelets enable multi-resolution analysis, allowing fishery time series data to be examined at different scales, identifying patterns and structures, and providing deeper insights into the underlying processes **(Lan et al., 2021)**.  3. Wavelet transforms effectively handle noise and non-stationarity in real-world time series fishery data by identifying and filtering out noise, thereby improving the understanding of the underlying signal **(Wu et al., 2022)**.  4. Wavelet coherence aids in comparing time series, like environmental indices and biological populations, by identifying time-localized correlation structures, providing a nuanced understanding of evolution over time **(Yadav et al., 2020)**.  **Brief description is added to the discussion section as follows:**  “Wavelet analysis is beneficial for analyzing simple time series fishery data due to its time-frequency localization, multi-resolution analysis, handling noise and non-stationarity, and its ability to compare time series like environmental indices and biological populations. It allows for the identification of transient features, patterns, and structures, providing deeper insights into underlying processes. Wavelet coherence aids in comparing time series like fishery and climatic oscillations data, providing a nuanced understanding of evolution over time.” **Line 805-813** |
| 4. Add unit to Period in all wavelet Figs .. also enlarge the font size | We would like the thank the reviewer for this important comment. Figures are rectified now. |

**Comment-wise Replies to Reviewer 2:**

| **Comments** | **Replies** |
| --- | --- |
| Lines141-142: Please also add the species descriptor | We would like the thank the reviewer for this important comment. Descriptor is now added in the revised manuscript as follows:  “The striped marlin, a renowned predator in recreational fishing, is known for its elongated body, vivid blue and silver hues, and large dorsal fin. Its vertical stripes become more prominent when stimulated or engaged in hunting. The blue marlin, with its vibrant cobalt blue upper body and shiny white underbelly, is a large predator with a long, pointed beak and a tall, curved dorsal fin. Its main food is fish and cephalopods. The silver marlin, with its streamlined, elongated body, cobalt blue dorsal surface, and pristine silvery ventral region, is a sought-after species for its dexterity and swiftness. The Atlantic white marlin's dorsal fin is conspicuous and curved, contributing to its sleek and streamlined appearance. The species primarily feeds on tiny fish and squid, making it an effective predator in its marine environment. These species are highly valued in recreational fishing for their dexterity and robustness.” **Line 141-152**. |
| Lines 155-157 and 157-160: please add references | We would like the thank the reviewer for this important comment. Following references are added now.  1. Sorenson, L., McDowell, J.R., Knott, T. and Graves, J.E. Assignment test method using hypervariable markers for blue marlin (Makaira nigricans) stock identification. Conservation Genetics Resources, 5, pp.293-297, https://doi.org/10.1007/s12686-012-9747-x, 2013.  **Ref 53**.  2. Conley, K.R. and Sutherland, K.R. Commercial fishers' perceptions of jellyfish interference in the Northern California Current. ICES Journal of Marine Science, 72(5), pp.1565-1575, https://doi.org/10.1093/icesjms/fsv007, 2015.  **Ref 18**.  3. Restrepo, V., Diaz, G. A., & Cortes, E. Stock assessment of Atlantic striped marlin (Kajikia audax) using catch data and abundance indices from commercial and recreational fisheries. ICES Journal of Marine Science, 68(9), 1884-1893. https://doi.org/10.1093/icesjms/fsp136, 2009. **Ref 49**.  4. Mamoozadeh, N. R., Graves, J. E., Bealey, R., Schratwieser, J., Holdsworth, J. C., Ortega-Garcia, S., & McDowell, J. R. Genomic data resolve long-standing uncertainty by distinguishing white marlin (Kajikia albida) and striped marlin (K. audax) as separate species. ICES Journal of Marine Science, 80(6), 1802-1813. https://doi.org 10.1093/icesjms/fsad136, 2023**. Ref 39**. |
| Lines 155-157: I’d suggest adding references here | We would like the thank the reviewer for this important comment. Following references are added now.  1. Sorenson, L., McDowell, J.R., Knott, T. and Graves, J.E. Assignment test method using hypervariable markers for blue marlin (Makaira nigricans) stock identification. Conservation Genetics Resources, 5, pp.293-297, https://doi.org/10.1007/s12686-012-9747-x, 2013.  **Ref 53**.  2. Conley, K.R. and Sutherland, K.R. Commercial fishers' perceptions of jellyfish interference in the Northern California Current. ICES Journal of Marine Science, 72(5), pp.1565-1575, https://doi.org/10.1093/icesjms/fsv007, 2015.  **Ref 18**. |
| Line 605: “vice versa” it should probably be in italic | Rectified now. **Line 606** |
| Lines 663-666: I would suggest spending few more words on how the authors are planning to investigate these aspects | We would like the thank the reviewer for this important comment. More description is added now as follows:  “The authors planned to study the relationship between climatic oscillations and marlin life phases using a multi-faceted approach. They will collect extensive data on climatic patterns, and cross-reference it with historical and contemporary biological records of marlin species. They will use advanced statistical models and machine learning algorithms to analyze the correlations between climatic oscillations and marlin life phases, identifying potential lag effects and understanding the resilience and adaptability of marlin species to changing environmental conditions. The study will also include a geographical analysis to identify regional variations in the impact of climatic oscillations. The findings are expected to have significant implications for marine conservation strategies, helping to predict and mitigate climate change impacts on marlin populations and ensuring their sustainability for future generations.” **Line 666-677**. |
